# A Comparative Study of Parameter Identification Methods for Asymmetric Nonlinear Systems with Quadratic and Cubic Stiffness

**DOI:** 10.3390/s22155854

**Published:** 2022-08-05

**Authors:** Shibo Wang, Bin Tang

**Affiliations:** 1 Key Laboratory of Ocean Energy Utilization and Energy Conservation of Ministry of Education, Dalian University of Technology, Dalian 116023, China; 2 Institute of Internal Combustion Engine, Dalian University of Technology, Dalian 116023, China

**Keywords:** quadratic and cubic stiffness nonlinearity, nonlinear system identification, envelope, instantaneous frequency, nonlinear vibration absorber

## Abstract

Understanding the nonlinear dynamic characteristics of engineering structures is challenging, especially for the systems that exhibit asymmetric nonlinear behavior. This paper compared four parameter identification methods for asymmetric nonlinear systems incorporating quadratic and cubic stiffness nonlinearities. Hilbert transform, zero-crossing, direct quadrature, and wavelet transform were used to obtain the backbone, envelope, and restoring force curves from the free vibration time history. A nonlinear curve-fitting method was then applied to estimate the stiffness parameters of the asymmetric systems, and a linear least square fitting approach was utilized to estimate the damping parameters of the asymmetric systems. We used the Helmholtz–Duffing oscillator as a numerical example and a nonlinear vibration absorber with geometric imperfections to verify the feasibility and accuracy of these methods. The advantages and disadvantages of these methods and the deviations in estimated results are discussed.

## 1. Introduction

Asymmetric nonlinear engineering structures, such as a mistuning quasi-zero-stiffness vibration isolator [1,2], cables [3], and geometric imperfect plate-like designs [4,5,6,7], have attracted widespread attention. The dynamic characteristics of these asymmetric systems are more complicated to analyze than those of the symmetric systems. For example, constant drift can occur in the response when there is little linear stiffness in the quasi-zero-stiffness vibration isolator. The nonlinear isolation system exhibits a mixed softening and hardening characteristic [1], which results in the multiple jump phenomena and hysteretic behavior [2]. Multivaluedness of the response curves occurs with different features depending on cables (or plates) and excitation force parameters [3,4,5,6,7]. To fully understand the nonlinear aspect of asymmetric systems, nonlinear parameter identification is one of the crucial procedures [8,9,10].

In recent years, backbone curves have been used to identify stiffness parameters of asymmetric systems. The calculation methods of the backbone curves can be classified into two types: analytical and numerical methods. Analytical methods include harmonic balance, multi-scale [11], and normal form methods [12,13]. The comparison of these analytical methods can be referred to [14]. Some software packages have implemented numerical algorithms based on a nonlinear normal mode framework [15,16,17]. Common experimental methods for extracting backbone curves include the resonance decay method [18], the control-based continuation method [19,20] and phase-locked loops [21]. For multi-degree-of-freedom systems, the nonlinear normal modes of interest are usually isolated by the force appropriation method [22,23,24]. The resonance decay method is then used to estimate the backbone curves. Breunung and Haller [25] recently studied the backbone curves of the forced-damped nonlinear mechanical systems. Cenedese and Haller [26] summarized approaches for constructing backbone curves of multi-degree-of-freedom systems. There are many applications to identify parameters using backbone curves, such as base-excited SDOF system [27], vibration absorber [28,29], beam-shaker system [30], aircraft wing structure [31], linear–arch composite beam piezoelectric energy harvester [32]. In addition to traditional contact measurement methods, non-contact measurement methods, such as video processing, can also be used to identify the backbone curves of an air wing prototype [33]. The restoring force curve is another useful tool that can be applied to estimate stiffness parameters of asymmetric systems [34]. Because of the asymmetric characteristics, the positive and negative parts of the asymmetric system were analyzed separately [35,36]. The bias term obtained by the signal decomposition method is the unique information in the asymmetric signal.

Hilbert transform [37], zero-crossing method [27], direct quadrature method [38], and wavelet transform [39] can be used to obtain the backbone curves and restoring force curves from the free-decay measurements. The applications of these methods to a Duffing system have been summarized [40,41]. However, these methods cannot be directly applied to asymmetric systems with quadratic and cubic stiffness nonlinearities, and need to be modified to analyze positive and negative time responses separately when obtaining the restoring force curves. The backbone curves exhibit softening-hardening nonlinear behavior and are complex to analyze. As far as the authors know, there is no relevant comparative study. Therefore, several identification methods are summarized and compared, which can better guide the understanding of the parameter identification of Helmholtz–Duffing type asymmetric engineering structures.

This paper aims to investigate the parameter identification methods applied to asymmetric systems with quadratic and cubic stiffness nonlinearities and illustrate these methods using a Helmholtz–Duffing numerical example and a vibration absorber experiment. Section 2 introduces the Helmholtz–Duffing example and its theoretical solutions for restoring force and backbone curves. In Section 3, detailed steps of the four methods to obtain different curves are introduced. Section 4 discusses the identification methods, identification results, errors of the four methods, and the corresponding advantages and disadvantages. The application in the vibration absorber experiment is described in Section 5. Finally, this paper is concluded in Section 6.

## 2. Asymmetric Model: A Helmholtz–Duffing Oscillator

The asymmetric engineering structures can be simplified to a Helmholtz–Duffing oscillator, and its equation of motion is given by

(1) $$m\ddot{x}+c\dot{x}+k_{1}x+k_{2}x^{2}+k_{3}x^{3}=f(t)$$

where *m* is the mass, *c* is the damping coefficient, *f*(*t*) is the excitation force, *k*_1_, *k*_2_, and *k*_3_ are the linear, quadratic, and cubic nonlinear stiffness coefficients, respectively. If the system vibrates freely with an initial displacement, *f*(*t*) = 0. Then, dividing both sides of Equation (1) by the mass *m* yields

(2) $$\ddot{x}+2h\dot{x}+\omega_{0}^{2}(t)x=0$$

where h=c/2m is the damping factor and $\omega_{0}(t)=\sqrt{\left(k_{1}+k_{2}x+k_{3}x^{2}\right)/m}$ is the instantaneous frequency. They have the units of 1/s. It can be seen that the analytical solution for the viscous damping force per unit mass is written as $f_{c}(x)=2h\dot{x}$ when the range of velocity is given, and the analytical solution for the restoring force per unit mass is given by

(3) $$f_{k}(x)=\frac{k_{1}}{m}x+\frac{k_{2}}{m}x^{2}+\frac{k_{3}}{m}x^{3}$$

where the regime of displacement is determined.

Backbone curves for the Helmholtz–Duffing oscillator are more complex than those for the symmetric systems [42]. Using the harmonic balance method and substituting the approximate harmonic solution $x(t)=A_{0}+A_{1}\cos(\omega t+\theta )$ into Equation (1) yield

(4) $$\omega^{2}=\frac{k_{1}}{m}+\frac{2k_{2}}{m}A_{0}+\frac{3k_{3}}{m}A_{0}^{2}+\frac{3k_{3}}{4m}A_{1}^{2}$$

where $A_{0}={\left(\frac{f_{0}}{2k_{3}}+{\left(\frac{f_{0}^{2}}{4k_{3}^{2}}+{\left(\frac{A_{1}^{2}}{2}+\frac{\beta }{3k_{3}}\right)}^{3}\right)}^{1/2}\right)}^{1/3}+{\left(\frac{f_{0}}{2k_{3}}-{\left(\frac{f_{0}^{2}}{4k_{3}^{2}}+{\left(\frac{A_{1}^{2}}{2}+\frac{\beta }{3k_{3}}\right)}^{3}\right)}^{1/2}\right)}^{1/3}-\frac{k_{2}}{3k_{3}}$ is the bias term, $A_{1}^{2}=-\left(k_{3}A_{0}^{3}+k_{2}A_{0}^{2}+k_{1}A_{0}\right)/\left(3k_{3}A_{0}/2+k_{2}/2\right)$ is the first harmonic term, $\beta =k_{1}-k_{2}^{2}/3k_{3}$, and $f_{0}=k_{1}k_{2}/3k_{3}-2k_{2}^{3}/27k_{3}^{2}$. Adding more harmonic terms to the approximate harmonic solution can yield a more accurate solution, but the calculation of the backbone curve is complicated. Substituting the approximate harmonic solution $x(t)=A_{0}+A_{1}\cos(\omega t+\theta )+A_{2}\cos(2\omega t+\varphi )$, where the second harmonic term *A*_2_ is included, into Equation (1) for the lightly damped case, gives

(5) $$\omega^{2}\approx \frac{k_{1}}{m}+\frac{2k_{2}}{m}A_{0}+\frac{3k_{3}}{m}A_{0}^{2}+\frac{3k_{3}}{4m}A_{1}^{2}+\frac{k_{2}}{m}A_{2}+\frac{k_{3}}{m}\left(\frac{3}{2}A_{2}^{2}+3A_{0}A_{2}\right)$$

which can be compared well with the numerical results of the backbone curves [42]. The comparison between analytical solutions and the numerical results is shown in Section 3.

## 3. Identification Methods for the Characteristic Curves

This section introduces four methods for obtaining the characteristic curves of the Helmholtz–Duffing oscillator, which are restoring force curves, damping force curves, envelopes, and backbone curves. These curves are combined with the analytical solutions given in Section 2 to estimate the stiffness and damping parameters of asymmetric structures. The details are discussed in Section 4 and Section 5. For the sake of simplicity, the parameters for the oscillator are *m* = 0.1 kg, *c* = 0.4 Ns/m, *k*_1_ = 4000 N/m, *k*_2_ = −10^7^ N/m^2^, and *k*_3_ = 10^10^ N/m^3^. The initial displacement and velocity are x(0)=0.0018 m and $\dot{x}(0)=0\;m/s$. The free-decay response is numerical integration calculated by using the fourth-order Runge-Kutta method, and the sampling frequency is *f*_s_ = 2000 Hz.

For the asymmetric system, when the free decay response is measured, the restoring force curve is constructed separately from the positive and negative signal parts [35], which is given by

(6) $$f_{k}(x)=\{\begin{array}{cc} \omega_{c}^{2}(t)A_{c}(t) & x>0\\ \omega_{c}^{2}(t)A_{c}(t) & x\leq 0 \end{array}$$

where $A_{c}$ and $\omega_{c}$ included positive and negative congruent envelopes and congruent natural frequency. The viscous damping force is approximately given by

(7) $$f_{c}(x)\approx \{\begin{array}{cc} 2h(t)A_{\dot{x}}(t) & \dot{x}>0\\ -2h(t)A_{\dot{x}}(t) & \dot{x}<0 \end{array}$$

where h(t) is the instantaneous damping factor and $A_{\dot{x}}(t)$ is the congruent envelope of velocity. The backbone curves can be obtained using the relationship between instantaneous frequency and amplitude of each harmonic term.

### 3.1. Hilbert Transform

Hilbert transform (HT) has been widely used in the parameter identification and signal decomposition of nonlinear systems. Feldman applied this method to identify free and forced vibration systems, and further applied the nonparametric identification method to the asymmetric systems [43,44,45,46]. The complex analytic form of a free-decay response is given by $X(t)=x(t)+j\tilde{x}(t)=A(t)e^{j\varphi (t)}$, where $\tilde{x}(t)$ is the Hilbert transform of the signal x(t), the envelope $A(t)=\sqrt{x^{2}(t)+{\tilde{x}}^{2}\left(t\right)}$, and the instantaneous phase $\varphi (t)=\arctan\left[\tilde{x}(t)/x(t)\right]$. The instantaneous undamped natural frequency is given by

(8) $$\omega_{0}^{2}(t)=\omega^{2}-\frac{\ddot{A}}{A}+\frac{2{\dot{A}}^{2}}{A^{2}}+\frac{\dot{A}\dot{\omega }}{A\omega }$$

and the instantaneous damping factor is given by

(9) $$h(t)=-\frac{\dot{A}}{A}-\frac{\dot{\omega }}{2\omega }$$

where $\dot{A}$, $\ddot{A}$ and $\dot{\omega }$ are the first and second derivatives of envelope and frequency, respectively.

Hilbert vibration decomposition (HVD) is a time-varying vibration decomposition method based on the Hilbert transform [45]. The main harmonic components of asymmetric systems can be obtained by using the HVD. By weighted summing the decomposed harmonic components, the congruent envelope, and the congruent frequency can be obtained. The congruent envelope $A_{c}$ is given by

(10) $$A_{c}(t)=\sum_{l=1}^{N}A_{l}(t)\cos\phi_{l}(t)$$

where *A_l_* is the envelope of the *l*th order component, and $\phi_{l}$ is the phase angle between the primary component and *l*th order component. Instantaneous natural frequency $\omega_{0}$ can be decomposed into a sum of high-order intrinsic components. The congruent natural frequency $\omega_{c}$ is given by

(11) $$\omega_{c}(t)=\sum_{l=1}^{N}\omega_{0l}(t)\cos\phi_{\omega l}(t)$$

where $\omega_{0l}$ is the envelope of the *l*th order instantaneous natural frequency, and $\phi_{\omega l}$ is the phase angle between the primary component and *l*th order component.

**Figure 1:**
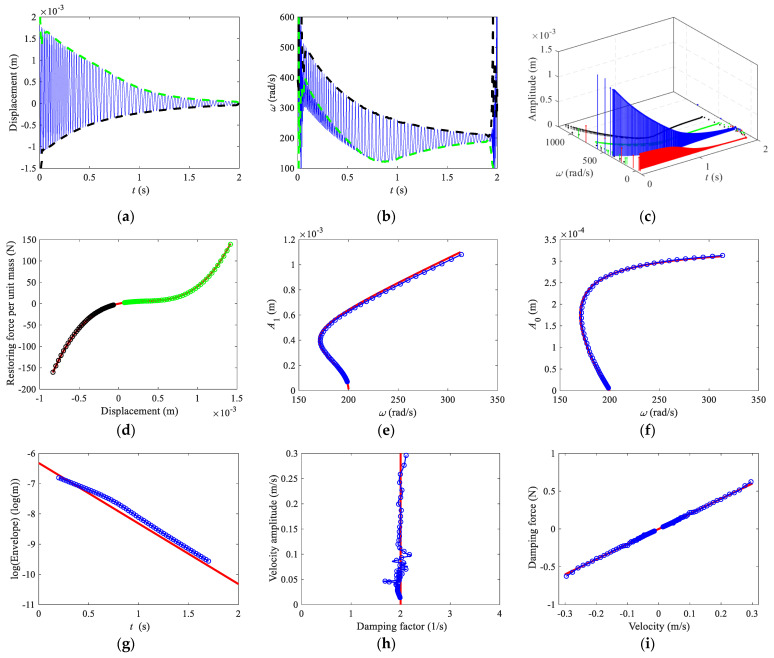
Hilbert transform. (**a**) Time domain response and envelopes, (**b**) frequencies, (**c**) HVD, (**d**) restoring force curve, (**e**) backbone curve of first harmonic term *A*_1_, (**f**) backbone curve of bias term *A*_0_, (**g**) logarithmic form of the envelope, (**h**) damping factor, (**i**) damping force curve. In (**a**,**b**), thin solid lines, time domain response, and instantaneous natural frequency; dashed lines, congruent envelope, and congruent modal frequency. In (**d**–**i**), solid lines, analytical solutions; lines with circles, numerical results.

The Hilbert transform method and the relevant Matlab programs [46] are used in this paper. Free-decay response of the Helmholtz–Duffing oscillator and its envelopes are shown in Figure 1a, where the positive and negative congruent envelopes are obtained by Equation (10). The instantaneous natural frequency and their envelopes are obtained by Equations (8) and (11), as shown in Figure 1b. Figure 1c shows the first fourth components of the free vibration obtained by using HVD. In order to remove the end effect of the Hilbert transform, only the analyzed results between 0.2 to 1.7 s are chosen. The restoring force curves are shown in Figure 1d, where the analytical solution is given by Equation (3), and the numerical results are obtained by Equation (6). The backbone curves of the first harmonic term *A*_1_ and bias term *A*_0_ are plotted in Figure 1e–f, respectively, where the analytical solutions are given by Equation (5), and the numerical results are obtained by using HVD. The logarithmic form of the envelope, instantaneous damping factor obtained by Equation (9), and damping force curve obtained by Equation (7) are shown in Figure 1g–i, respectively. For a weakly nonlinear system, the analytical solution of the logarithmic envelope is approximately given by −ct/2m+lnx(0), as shown in Figure 1g. The damping factor is *h* = *c*/2*m* = 21/s and the analytical damping force is $f_{c}(x)=2h\dot{x}$, which are plotted in Figure 1h,i, respectively.

### 3.2. Zero-Crossing

**Figure 2:**
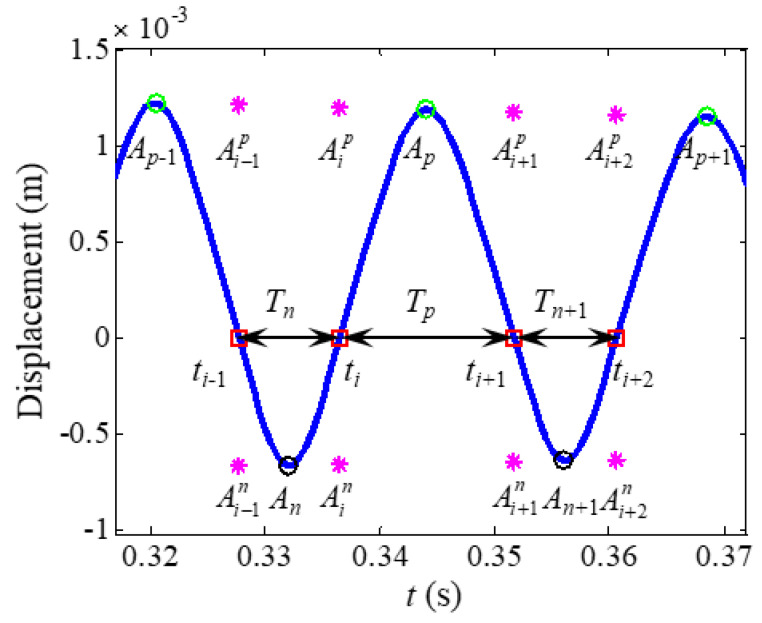
Zero-crossing with peak picking method for the asymmetric signal. Solid line, time-domain response; **□**, zero-crossing points; **◯**, positive peak points; **◯**, negative peak points; *, envelopes at zero-crossing points obtained by interpolation.

The zero-crossing (ZC) method is a simple method for estimating the instantaneous frequency and amplitude of free decay signals [27,36]. Londoño et al. [27] combined the zero-crossing and peak picking methods to obtain the backbone curves of symmetric nonlinear systems. Ondra et al. [36] extended the zero-crossing method to obtain the backbone and restoring force curves of asymmetric systems. This method is explained in detail in Figure 2. The *i*th zero-crossing point *t_i_*, the *p*th positive peak points *A_p_*, and the *n*th negative peak points *A_n_* are first obtained using the zero and peak picking procedure. The instantaneous frequency at the zero-crossing point *t_i_* is

(12) $$\omega (t_{i})=\frac{2\pi }{t_{i+1}-t_{i-1}}$$

The positive and negative envelopes at the zero-crossing points are then obtained by linear interpolating the positive and negative peak points, respectively. The corresponding instantaneous frequencies for positive and negative envelope points are *ω_p_* = *π*/*T_p_* and *ω_n_* = *π*/*T_n_*, respectively.

The amplitudes of the first harmonic term *A*_1_ and bias term *A*_0_ are given by

(13) $$A_{0}=\frac{A_{i}^{p}+A_{i}^{n}}{2},\;A_{1}=\frac{A_{i}^{p}-A_{i}^{n}}{2}$$

**Figure 3:**
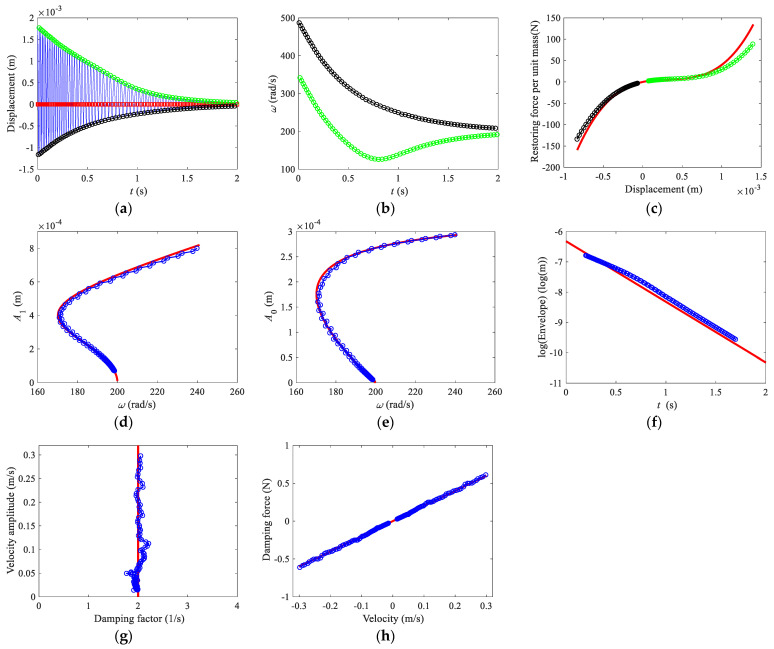
Zero-crossing with peak picking method. (**a**) Time domain response, (**b**) frequencies, (**c**) restoring force curve, (**d**) backbone curve of first harmonic term *A*_1_, (**e**) backbone curve of bias term *A*_0_, (**f**) logarithmic form of the envelope, (**g**) damping factor, (**h**) damping force curve. In (**a**,**b**), solid line, time domain response; red squares, zero-crossing points; lines with circles, positive and negative envelopes, and frequencies. In (**c**–**h**), solid lines, analytical solutions; lines with circles, numerical results.

The free decay response is given in Figure 3a again. Then, the zero-crossing points and positive and negative peak points can be obtained and plotted in Figure 3a. The instantaneous frequencies for the positive and negative envelopes are shown in Figure 3b. Substituting the positive and negative envelopes and instantaneous frequencies in Equation (6) yields the restoring force curves, as shown in Figure 3c. It should be noticed that the time history response of asymmetric systems normally contains multiple harmonic components. Unlike the HVD, the backbone curves in the zero-crossing method are calculated using Equations (12) and (13), where the high order harmonic terms induce estimation errors, especially for the bias term. Before calculating the instantaneous frequency and amplitudes, the high-order harmonic terms should be filtered to make sure that the response can be approximately written as $x(t)\approx A_{0}+A_{1}\cos(\omega t+\theta )$. For the numerical example in this section, the free decay signal is passed through a low-pass filter with a cut-off frequency of 50 Hz. The backbone curves are shown in Figure 3d,e. It can be seen that the backbone curves obtained from the filtered response are closer to the analytical backbone curves. The logarithmic form of the envelope, instantaneous damping factor, and damping force curve are shown in Figure 3f–h. Numerical results for the damping factor and damping force are obtained by Equations (7) and (9), where the envelope of velocity is obtained by the peak picking method. To unify several methods, the data used in the restoring force curve, logarithmic envelope, and damping curve are also approximately taken from 0.2 to 1.7 s. The data used in backbone curves are relatively short because part of the response in the large amplitude regime is filtered out. The analytical solutions shown in Figure 3 are obtained using the similar approaches mentioned in Section 3.1.

### 3.3. Direct Quadrature

The direct quadrature (DQ) method was proposed by Huang et al. [38]. Firstly, because the Hilbert transform of a product of functions is limited by the Bedrosian theorem [47], a normalization scheme was proposed to separate amplitude modulation (AM) and frequency modulation (FM) of the signal. Secondly, according to the Nuttall theorem [48], it is not applicable for all signals to obtain their quadrature forms using the Hilbert transform. Therefore, the direct quadrature method is used. The direct quadrature method has been applied to the symmetric signal. However, when it is applied to the asymmetric signal, some modifications are required.

The first step is to find the positive and negative peak points and use the cubic spline function to obtain the positive and negative envelopes *A_p_* and *A_n_*. Then the normalization process is carried out. The positive time-domain response is normalized by $y_{p}(t_{p})=x_{p}(t_{p})/A_{p}$ and the negative time-domain response is normalized by $y_{n}(t_{n})=x_{n}(t_{n})/A_{n}$, where $x_{p}(t_{p})$ and $x_{n}(t_{n})$ are the positive and negative time-domain responses, $y_{p}(t_{p})$ and $y_{n}(t_{n})$ are the normalized positive and negative responses. Repeat the above steps until the normalized results are all in [−1,1]. The FM part of the signal is $F(t)=y^{l}(t)=\left[y_{p}^{l}(t_{p}),y_{n}^{l}(t_{n})\right]$, where *l* is the number of iterations.

After normalization, the AM part is given by

(14) $${\tilde{A}}_{p}(t_{p})=\frac{x_{p}(t_{p})}{y_{p}^{l}(t_{p})},\;{\tilde{A}}_{n}(t_{n})=\frac{x_{n}(t_{n})}{y_{n}^{l}(t_{n})}$$

Then using the cubic spline function, the positive envelope ${\tilde{A}}_{p}(t)$ and negative envelope ${\tilde{A}}_{n}(t)$ in the entire time domain are obtained. The amplitudes of the first harmonic and the bias terms are given by

(15) $$A_{1}=\frac{{\tilde{A}}_{p}(t)-{\tilde{A}}_{n}(t)}{2},\;A_{0}=\frac{{\tilde{A}}_{p}(t)+{\tilde{A}}_{n}(t)}{2}$$

**Figure 4:**
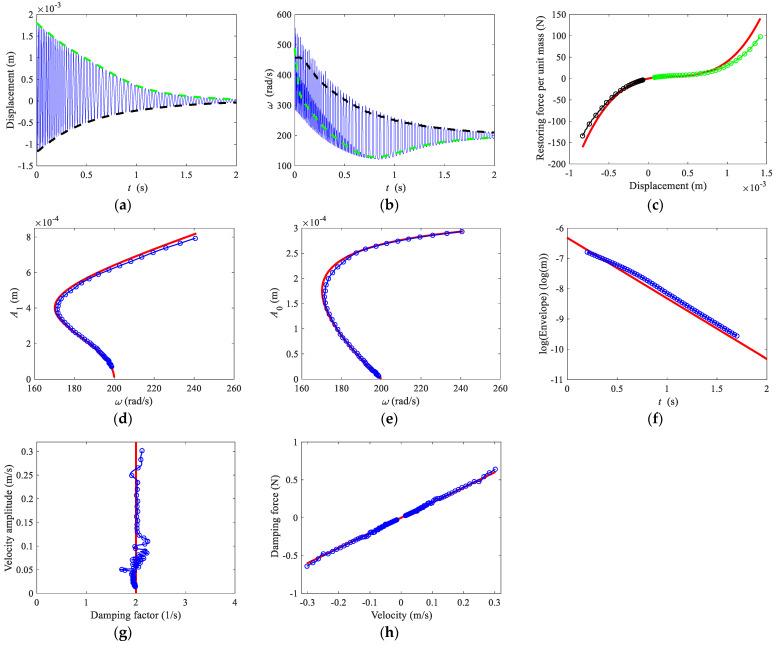
Direct quadrature. (**a**) Time domain response and envelopes, (**b**) frequencies, (**c**) restoring force curve, (**d**) backbone curve of first harmonic term *A*_1_, (**e**) backbone curve of bias term *A*_0_, (**f**) logarithmic form of the envelope, (**g**) damping factor, (**h**) damping force curve. Thin solid lines, time domain response in (**a**) and instantaneous frequency in (**b**); dashed lines, positive and negative envelopes in (**a**) and frequencies in (**b**). In (**c**–**h**), thick solid lines, analytical solutions; lines with circles, numerical results.

The time domain response and its positive and negative envelopes are shown in Figure 4a. The FM part can be regarded as a sinusoid, so the instantaneous frequency is

(16) $$\omega (t)=\frac{d}{dt}\left[\arccos(F(t))\right]$$

The FM part whose absolute values are less than 0.9 is used to calculate the instantaneous frequency using Equation (16), and the rest of the instantaneous frequency points are interpolated using a cubic spline. The result is shown in Figure 4b. The restoring force curve obtained by Equation (6) is shown in Figure 4c. Similar to the zero-crossing method, the amplitudes of the first harmonic and the bias terms are also sensitive to the high-frequency components of the free decay response. Before calculating the backbone curves, the free decay response is passed through a low-pass filter with a cut-off frequency of 50 Hz. The estimated backbone curves of *A*_1_ and *A*_0_ are shown in Figure 4d,e, where amplitudes are obtained by Equation (15), and the frequency is the filtered instantaneous frequency. The logarithmic form of the envelope, instantaneous damping factor, and damping force curve are shown in Figure 4f–h. The analytical solutions are also shown in Figure 4.

### 3.4. Wavelet Transform

Wavelet transform (WT) is a time-frequency analysis tool that can automatically adjust the size of the analysis window with the change of frequency. With the development in recent years, wavelet analysis has been widely applied in nonlinear system identification [39,49,50,51].

**Figure 5:**
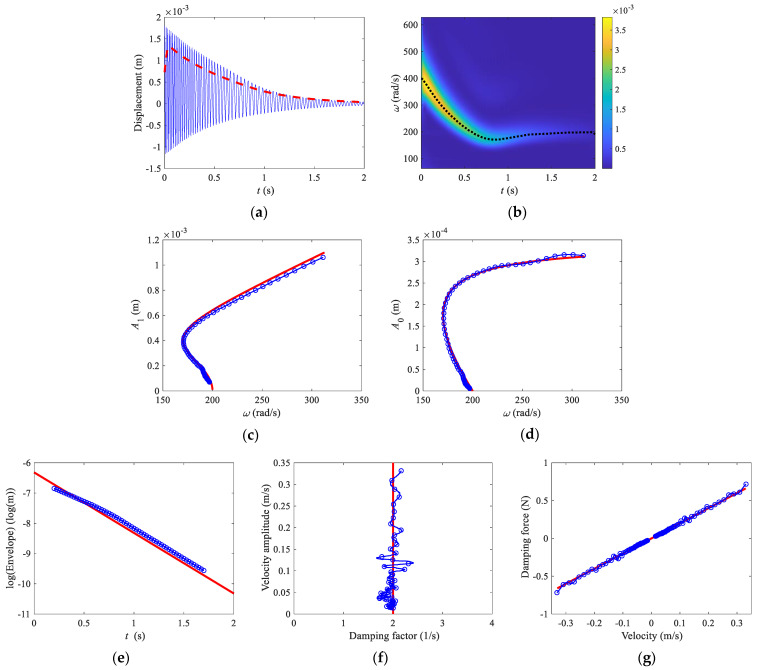
Wavelet transform. (**a**) Time-domain response and envelope, (**b**) spectrogram, (**c**) backbone curve of first harmonic term *A*_1_, (**d**) backbone curve of bias term *A*_0_, (**e**) logarithmic form of the envelope, (**f**) damping factor, (**g**) damping force curve. Thin solid line, time domain response; dashed line, envelope; dotted line, instantaneous frequency; thick solid lines, analytical solutions; lines with circles, numerical results.

For the free decay response of the Helmholtz–Duffing oscillator shown in Figure 5a, the frequency spectrum can be obtained from the Matlab *cwt* function using the Morlet wavelet and is shown in Figure 5b. From this figure, the envelope of the signal is given by

(17) $$A(b)=\frac{2\left|W_{x}(a(b),b)\right|}{\sqrt{a(b)}}$$

where *a* is the scale parameter, *b* is the translation parameter, $\left|W_{x}(a(b),b)\right|=\max_{a}\left|W_{x}(a,b)\right|$ is the maximum value of wavelet coefficients at each time point. The instantaneous frequency is obtained from the frequency points corresponding to the maximum value of the wavelet coefficients at each time point. In order to obtain the smooth envelope and instantaneous frequency, the results are passed through a low-pass filter with a cut-off frequency of 20 Hz. The results are shown in Figure 5a,b. The backbone curve of *A*_1_ constructed by the instantaneous amplitude and frequency is shown in Figure 5c. The bias term is obtained by the wavelet decomposition using the Matlab function *wavedec*, as shown in Figure 5d. The logarithmic form of the envelope, instantaneous damping factor, and damping force curve are shown in Figure 5e–g. It can be seen that the numerical solutions can be reasonably compared well with the analytical solutions.

## 4. Parameter Estimation and Discussion

In this section, the stiffness and damping parameters of the asymmetric systems are estimated from the characteristic curves of the Helmholtz–Duffing oscillator obtained in Section 3. The stiffness parameters are obtained by polynomial fitting the restoring force curve shown in Equation (3). The Matlab function *polyfit* computes the least square polynomial. This method is called the restoring force curve method (RFCM). Although the backbone curve of the first harmonic term obtained by using Equation (4) deviates from the numerical result obtained by using HVD in the bending regime [42], which is the curved part, we can also estimate the stiffness parameters from this curve. Matlab function *fminsearch* is used here to find the optimal stiffness parameters. This identification method is called the backbone curve method (BCM). The estimated stiffness parameters are shown in Table 1.

The viscous damping can be estimated by linear fitting the damping force curve, called the damping force curve method (DFCM). For a weakly nonlinear system, the envelope is approximately given by $\ln A(t)=-ct/2m+\ln A_{0}$. Therefore, the natural logarithm of the envelope can also be used to estimate the damping [28], called the logarithmic envelope method (LEM). Matlab function *polyfit* is utilized for computing the least square linear coefficient. The estimated damping coefficients are shown in Table 2.

The estimated stiffness parameters are obtained by using the four methods. The Hilbert transform and Hilbert vibration decomposition combined with the low pass filter can give accurate estimation results. This method can not only decompose the signal and obtain the backbone curve of each harmonic term, but also combine harmonic terms to construct the restoring force. However, the Hilbert transform method has an end effect. The data at the beginning and end need to be removed. For zero-crossing with the peak picking method, it is straightforward to implement. Even for the asymmetric signal, this method can analyze the positive and negative time domain separately and construct the corresponding restoring force. However, when this method is applied to the signal contaminated with noise, the zero-crossing points and peak points are difficult to obtain accurately. The signal should be properly filtered to solve this issue. Also, there are only fewer points to extract for the short-time signal. In this case, the interpolation method can be used in the whole time domain to obtain more points. For the direct quadrature method, part of instantaneous frequency points are interpolated using a cubic spline, so the obtained instantaneous frequency is not accurate enough, especially for the signal with a low sampling rate or a large amount of normalized data is over 0.9. For the wavelet transform, the wavelet function needs to be selected carefully and appropriately.

The nonlinear stiffness parameters estimated by the restoring force curve using the zero-crossing and direct quadrature methods are not accurate. The estimated results of the backbone curve method are all well. The backbone curve method estimates the entire backbone curve, so the estimated stiffness parameters are comprehensively affected by the deviation of each amplitude regime.

For the damping coefficient, the four methods seem to achieve similar results. The estimated solutions of the logarithmic envelope method are all less than the actual value because it uses the analytical solution of the linear system. But this method is simple and easy to estimate for weakly damped systems. The error of the damping force curve method is small. The derivatives of the envelope, instantaneous frequency, and displacement should be obtained for the damping force curve method, so the result is disturbed by noise easily. A proper filter can be used to deal with the influence of the noise, and the Bayesian approach is a good way to measure the uncertainty of the identification results [29].

## 5. Experiment

### 5.1. Experimental Description

**Figure 6:**
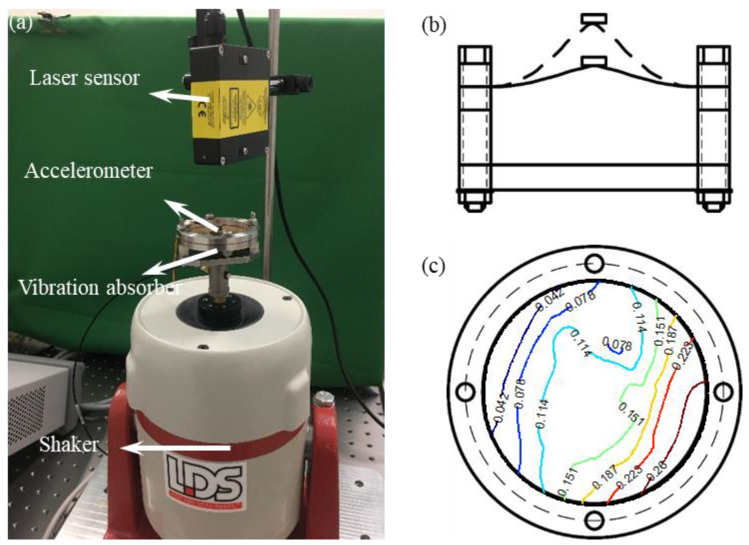
Test rig and schematic model of the vibration absorber. (**a**) Photo of the test rig, (**b**) elevation view, (**c**) Plan view and contour plot for the measured geometric imperfections. Relative deviations are in millimeters.

The test rig is shown in Figure 6, where Figure 6a is the photo of the test rig, and Figure 6b,c are the elevation and plan views of the nonlinear vibration absorber. The vibration absorber consists of a 4.86 g mass attached to a thin circle brass plate of 0.15 mm thickness. Figure 6c also shows the contour plot for the measured geometric imperfections obtained by moving the laser sensor through the translation surface of the plate. It can be seen that the plate is not flat, and has a certain initial deflection, as shown in Figure 6b.

The excitation signal was generated by the LDS V406 shaker, then measured by the B&K 4517 accelerometer and Microtrak™ 3 LTS-050-10 laser sensor, respectively. The sampling frequency was 2000 Hz. Because the linear natural frequency of the vibration absorber was much higher than the natural frequency of the shaker, the absorber and the shaker can be regarded as a single-degree-of-freedom system [28]. The excitation signal supplied to the shaker was from Agilent 33512B signal generator and passed through an LDS PA500L power amplifier. The measured signals were sampled by a NI PXIe-4492 acquisition system after passing through a B&K 2693 conditioner. The equation of motion of the plate with geometric imperfections can be simplified as a Helmholtz–Duffing oscillator, as discussed in [4,28]. Therefore, the mathematical model of the free-decay response of the nonlinear vibration absorber is given by

(18) $$m_{\mathrm{EQ}}\ddot{x}+c\dot{x}+k_{1}x+k_{2}x^{2}+k_{3}x^{3}=0$$

where $m_{\mathrm{EQ}}=\left(m_{s}+m_{v})(m_{a}+m\right)/\left(m_{s}+m_{v}+m_{a}+m\right)=5.44\;g$ is the equivalent mass of the system. *m_s_*, *m_v_*, *m_a_*, and *m* are the mass of armature, support structure of the absorber, accelerometer, and absorber mass, respectively. *x* is the vibration response of the experimental system. Damping *c*, linear stiffness *k*_1_, quadratic and cubic nonlinear stiffness *k*_2_ and *k*_3_ are the parameters to be estimated.

### 5.2. Estimation and Discussion

**Figure 7:**
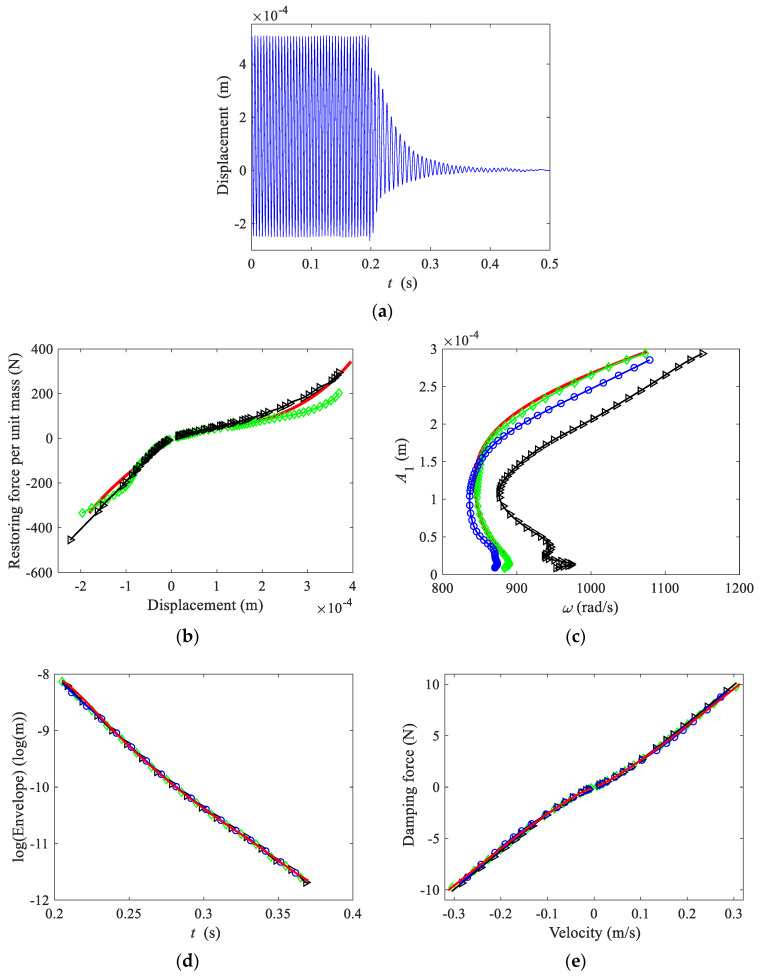
Measured (**a**) time domain response, (**b**) restoring force curve, (**c**) backbone curve, (**d**) logarithmic form of the envelope, (**e**) damping force curve. Thin solid line, time domain response; thick solid lines, Hilbert transform; lines with diamonds, zero-crossing; lines with triangles, direct quadrature; lines with circles, wavelet transform.

Before the free decay experiment, several slow frequency sweep experiments from low to high frequency were carried out to determine the system’s jump-down frequency, which was about 201 Hz. Therefore, the excitation signal was switched off at 200 Hz. The circular plate exhibited large stiffness nonlinearity, and the vibration modes of the system were well-separated. In order to exclude the influence of the higher harmonics and high order modes, the displacement of the mass measured by the laser sensor was passed through a low pass filter with a cut-off frequency of 270 Hz, as shown in Figure 7a. The specific experimental instruments and testing procedures were described in [42]. The data used in identification is approximately taken from 0.205 to 0.37 s.

Figure 7b shows the restoring force curve. Because the response decayed fast, cubic spline interpolation was used to construct envelope and frequency at each time point for the zero-crossing method. The first several large-amplitude points were removed due to the deviation. The restoring force was estimated by using Equation (3). The estimated stiffness parameters are shown in Table 3. The estimated results of the zero-crossing and direct quadrature methods are close to each other.

Figure 7c shows the backbone curve. The backbone curve obtained using the Hilbert transform is compared well with the backbone curve obtained using the zero-crossing method. The backbone curve obtained by using the wavelet transform shows a slight deviation from the above two methods, while the backbone curve obtained by using the direct quadrature method shows the most significant deviation. The estimated stiffness parameters are shown in Table 3. It can be seen that, except for the direct quadrature method, the estimated results of other methods are compared well. The identification results of the Hilbert transform can be used to reasonably reconstruct the experimental system’s restoring force and backbone curves. The stiffness parameters are also estimated well by the zero crossing method and wavelet transform using the backbone curve.

The natural logarithm of the envelope and the damping force curve are shown in Figure 7d,e. The results of the four methods are similar, as shown in Table 4. It can be seen that the damping coefficient estimated by the logarithmic envelope method is less than that estimated by the damping force curve method. The logarithmic envelope method uses the analytical solution of the envelope of the linear system. However, the damping of the experimental system is not strictly linear. It can be noticed from the logarithmic envelope and damping force curve that the damping factor is amplitude dependant and decreases with time.

To determine whether the identification procedure is successful, a reconstructed free decay response is compared with the measured response as shown in Figure 7a. The approximate solution of Equation (18) is given by

(19) $$x(t)=A_{0}(t)+A_{1}(t)\cos\left[\phi (t)\right]$$

where $A_{0}(t)$ is the time-dependent amplitude (or envelope) of bias term, $A_{1}(t)$ is the time-dependent amplitude of the first harmonic term, and ϕ(t) is the time-dependent phase. The time-dependent phase is obtained by integrating the time-dependent damped natural frequency

(20) $$\phi (t)=\int_{0}^{t}\omega (t)dt$$

**Figure 8:**
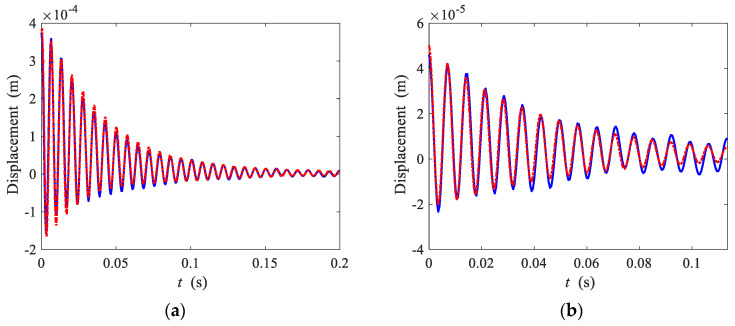
Experimental and reconstructed time domain response for (**a**) large initial displacement, and (**b**) small initial displacement. Solid lines, measured response; dashed-dotted lines, reconstructed response.

The time-dependent damped natural frequency, namely the backbone curve, is given by Equation (4). The parameters obtained by the Hilbert transform method using the backbone curve and the measured envelopes are used in reconstructing the free decay response. The results are shown in Figure 8a.

For the small amplitude regime, using the multiple-scales method [11], the time domain response is approximately given by

(21) $$x(t)=A_{0}(t)+A_{1}e^{-\zeta \omega_{n}t}\cos\left[\omega_{n}t+\frac{3}{16\zeta }\frac{k_{3}}{k_{1}}A_{1}^{2}(1-e^{-2\zeta \omega_{n}t})\right]$$

where $A_{0}(t)$ is the measured envelope of bias term, *A*_1_ is the initial amplitude of the first harmonic term. $\omega_{n}=\sqrt{k_{1}/m_{\mathrm{EQ}}}$ and $\zeta =c/2\sqrt{m_{\mathrm{EQ}}k_{1}}$ are the undamped natural frequency and the damping ratio of the underlying linear system respectively. Damping obtained by the logarithmic envelope method is used in reconstructing the time response. The results are shown in Figure 8b. It can be seen that the reconstructed responses obtained by the two reconstruction methods are compared well with the measured responses. The reconstruction method is described in detail in [28], which is applied to identify a Duffing type nonlinear vibration absorber.

## 6. Conclusions

This paper compared four identification methods for the stiffness and damping parameters of asymmetric systems with square and cubic nonlinearities. We verified the feasibility and accuracy of these methods by a Helmholtz–Duffing numerical example and a nonlinear vibration absorber with geometric imperfections. Hilbert vibration decomposition decomposes the asymmetric signal to obtain the backbone curve of each harmonic term. The asymmetric restoring force curve is constructed by the positive and negative congruent envelopes and frequencies obtained by using a weighted summing of the decomposed harmonic components. The obtained curves compare well with the analytical solution, but the disadvantage of the Hilbert transform is the end effect. Zero-crossing with the peak picking method extracts positive and negative peak points and obtains instantaneous frequency more simply. The bias term and first harmonic term are obtained by using the sum and difference of the positive and negative envelopes. The direct quadrature method also analyzes the positive and negative signal parts separately and uses the same method as the zero-crossing method to obtain the bias term and the first harmonic term. The two methods are more sensitive to noise. It is essential for the wavelet transform to select wavelet function appropriately.

The nonlinear stiffness parameters estimated are not accurate in the numerical example when the restoring force curve is obtained using the zero-crossing method and direct quadrature method. The identification errors for nonlinear stiffness parameters are about 20%. The other methods can estimate the stiffness parameters accurately, and the identification errors are less than 5%. For the experimental system, the identification results of the Hilbert transform can describe the experimental system. The stiffness parameters estimated by the restoring force curve using the zero-crossing method and the direct quadrature method are close to each other. The backbone curve obtained by using the direct quadrature method deviates from those obtained by the other methods.

The logarithmic envelope can be used to estimate damping. The estimated results are the same, and all are less than the actual values. The other tool is the damping force curve, which is constructed by the damping factor and envelope of the velocity. The estimated results are better than those of the logarithmic envelope. However, the damping force curve needs the derivative of the envelope, instantaneous frequency, and displacement, so it has poor robustness against noise. It can be seen from the logarithmic envelope and damping force curve that the damping of the experimental system is not strictly linear.

This paper is to identify the parameters of a predetermined system model. Restoring the force curve method and damping force curve method are nonparametric identification methods. They produce the best functional representation of the system without a priori assumption about the system model. However, it is necessary for the backbone curve method to know the nonlinear system model and the theoretical solution of the backbone curve in advance. The logarithmic envelope method is suitable for lightly damped systems and is an approximate estimation method.

## Figures and Tables

**Table 1:** Estimated results and errors of the stiffness parameters.

	*k*_1_ (N/m)		*k*_2_ (N/m^2^)		*k*_3_ (N/m^3^)	
	RFCM	BCM	RFCM	BCM	RFCM	BCM
HT	3940.7 (1.48%)	4082.0 (2.05%)	−1.002 × 10^7^ (0.20%)	−1.007 × 10^7^ (0.73%)	9.998 × 10^9^ (0.02%)	1.023 × 10^10^ (2.31%)
ZC	4051.5 (1.29%)	4050.6 (1.27%)	−8.381 × 10^6^ (16.19%)	−9.873 × 10^6^ (1.27%)	7.213 × 10^10^ (27.87%)	1.003 × 10^10^ (0.31%)
DQ	4052.5 (1.31%)	4066.5 (1.66%)	−8.273 × 10^6^ (17.27%)	−9.923 × 10^6^ (0.77%)	7.262 × 10^9^ (27.38%)	1.008 × 10^10^ (0.76%)
WT		3989.1 (0.27%)		−9.977 × 10^6^ (0.23%)		1.043 × 10^10^ (4.28%)
True value	4000		−10^7^		10^10^	

**Table 2:** Estimated results and errors of the damping coefficient.

*c* (Ns/m)	LEM	DFCM
HT	0.381 (4.80%)	0.402 (0.37%)
ZC	0.377 (5.76%)	0.406 (1.58%)
DQ	0.381 (4.82%)	0.408 (2.02%)
WT	0.370 (7.60%)	0.405 (1.30%)
True value	0.4	

**Table 3:** Estimated stiffness parameters of the experimental system.

	*k*_1_ (N/m)		*k*_2_ (N/m^2^)		*k*_3_ (N/m^3^)	
	RFCM	BCM	RFCM	BCM	RFCM	BCM
HT	4837.7	4272.9	−2.252 × 10^7^	−2.155 × 10^7^	5.705 × 10^10^	6.346 × 10^10^
ZC	5452.3	4265.7	−2.461 × 10^7^	−2.109 × 10^7^	4.822 × 10^10^	6.302 × 10^10^
DQ	5597.5	4949.9	−2.193 × 10^7^	−2.776 × 10^7^	5.123 × 10^10^	8.614 × 10^10^
WT		4137.9		−2.152 × 10^7^		7.096 × 10^10^

**Table 4:** Estimated damping coefficient of the experimental system.

*c* (Ns/m)	LEM	DFCM
HT	0.234	0.320
ZC	0.232	0.317
DQ	0.232	0.325
WT	0.230	0.311
